# Substitution treatment for opioid addicts in Germany

**DOI:** 10.1186/1477-7517-4-5

**Published:** 2007-02-02

**Authors:** Ingo Ilja Michels, Heino Stöver, Ralf Gerlach

**Affiliations:** 1 Head of the Office of the Federal Drug Commissioner, Federal Ministry of Health, Berlin, Germany (from 2006-2008: Shanghai/PR China); 2 Bremen Institute of Drug Research, University of Bremen, Germany; 3 Deputy Director, Institute for the Advancement of Qualitative Drug Research (INDRO), Münster, Germany

## Abstract

**Background:**

After a long and controversial debate methadone maintenance treatment (MMT) was first introduced in Germany in 1987. The number of patients in MMT – first low because of strict admission criteria – increased considerably since the 1990s up to some 65,000 at the end of 2006. In Germany each general practitioner (GP), who has completed an additional training in addiction medicine, is allowed to prescribe substitution drugs to opioid dependent patients. Currently 2,700 GPs prescribe substitution drugs. Psychosocial care should be made available to all MMT patients.

**Results:**

The results of research studies and practical experiences clearly indicate that patients benefit substantially from MMT with improvements in physical and psychological health. MMT proves successful in attaining high retention rates (65 % to 85 % in the first years, up to 50 % after more than seven years) and plays a major role in accessing and maintaining ongoing medical treatment for HIV and hepatitis. MMT is also seen as a vital factor in the process of social re-integration and it contributes to the reduction of drug related harms such as mortality and morbidity and to the prevention of infectious diseases. Some 10 % of MMT patients become drug-free in the long run. Methadone is the most commonly prescribed substitution medication in Germany, although buprenorphine is attaining rising importance. Access to MMT in rural areas is very patchy and still constitutes a problem. There are only few employment opportunities for patients participating in MMT, although regular employment is considered unanimously as a positive factor of treatment success. Substitution treatment in German prisons is heterogeneous in access and treatment modalities. Access is very patchy and the number of inmates in treatment is limited. Nevertheless, substitution treatment plays a substantial part in the health care system provided to drug users in Germany.

**Conclusion:**

In Germany, a history of substitution treatment spanning 20 years has meanwhile accumulated a wealth of experience, e.g. in the development of research on health care services, guidelines and the implementation of quality assurance measures. Implementing substitution treatment with concomitant effects and treatment elements such as drug history-taking, dosage setting, co-use of other psychoactive substances (alcohol, benzodiazepines, cocaine), management of 'difficult patient populations', and integration into the social environment has been arranged successfully. Also psychosocial counseling programmes adjuvant to substitution treatment have been established and, in the framework of a pilot project on heroin-based treatment, standardised manuals were developed. Research on allocating opioid users to the 'right' form of therapy at the 'right' point in time is still a challenge, though the pilot project 'heroin-based treatment' brought experience with patients who do not benefit from methadone treatment. There is also expertise in the treatment of specific co-morbidity such as HIV/AIDS, hepatitis and psychiatric disorders. The promotion and involvement of self-help groups plays an important part in the process of successful substitution treatment.

## Background

### Historical background

Heroin found its way onto the German illicit market around 1970 followed by a rapid increase in the number of heroin users and addicts. It is estimated that currently there are about 120,000 to 150,000 heroin users in Germany. Up to the mid 1980s, the national drug policy in Germany had been oriented towards the so called 'abstinence paradigm', but due to the rise of HIV-infections among injecting drug users the developments in legal, medical and political areas then changed towards a more pragmatic and harm-reduction oriented strategy [1-5]. Although the first experimental methadone project had already been carried out in Hanover in the mid 1970s, substitution treatment for heroin users remained a controversial issue in Germany for a very long time, because the study's conclusions were misguided by the majority of drug experts and politicians. Despite the fact that there was a 100 % reduction in criminal activities as well as social reintegration and vocational/occupational rehabilitation, the trial was deemed a failure because the patients failed to achieve and maintain abstinence [6].

On a larger scale this treatment option was introduced relatively late, primarily in response to the threat of the increasing prevalence of HIV and AIDS among injecting drug users (IDU) in Germany in the mid 1980s. However, it reflected rising public nuisance associated with drug use, increasing mortality rates among drug users, the lack of attractiveness of abstinence-oriented services and strong advocacy by a handful of dedicated parents of addicts in collaboration with an equally small number of GPs. These factors finally led to the implementation of harm-reduction-oriented services, i.e. low-threshold drop-in centres and syringe exchange schemes. The first large-scale methadone maintenance treatment programme (MMTP) was started in 1987 within the scope of a model project in one federal state (North-Rhine Westphalia). [7,8]

The German Narcotics Act was revised in 1992, finally clarifying that substitution treatment for opioid dependence is legal. Up to the present substitution treatment has been the most important part of the options available for the treatment of opioid dependence. Over the past 15 years the overall number of participants in drug-substitution treatment has risen from some 1,000 in the late 1980s to 65,000 in 2006 [9], and although MMT has been evaluated comprehensively in Germany with favourable outcomes there is still a lack of availability of, and accessibility to, substitution treatment. [10]

Until the early 1990s methadone could only be administered to drug users when highly specific indication criteria were met (e.g. emergency cases, such as life-threatening conditions of withdrawal, severe pain, pregnancy or HIV infection). However, there were a few general practitioners (GPs) who ignored the legal regulations and prescribed methadone to opiate addicts, but most of them were persecuted and prosecuted. Some GPs started prescribing codeine or dihydrocodeine (DHC) (provided in the form of juice) as these substances were not restricted by narcotic law [11]. Other doctors followed this example and over many years codeine or DHC came to be prescribed to very large numbers of addicts under a loop-hole in narcotics regulations.

After several pilot programmes showed MMT to be effective the German Social Health Insurers (SHI) approved this treatment modality and introduced, in 1991, methadone treatment guidelines for financing this kind of treatment. In Germany treatment and prescription (medication) costs are generally paid by public health insurance schemes (SHI) that provide legally mandated coverage for almost 90 percent of the population (in special cases, e.g. homelessness, doctors' fees are met by social welfare services). There is also the freedom to choose one's own general practitioner (GP) or hospital.

### Legal framework of substitution treatment

Since the 1920s, when the first Narcotics Act had been introduced in Germany, the main emphasis of legislation was placed on developing instruments for the control of the narcotic drugs trade rather than on measures of prevention, care, treatment and rehabilitation. Today, the purpose of the Narcotics Act is, above all, to ensure that there are sufficient supplies of licit narcotics for the medical care of the population (particularly for the treatment of severe conditions of pain), and, in addition, to minimize the likelihood of abuse of narcotic drugs and the emergence and maintenance of addiction as far as possible. Since 1981 increasing numbers of drug addicts and drug-dependent offenders led to an inclusion of detailed provisions of activities to reduce the demand for narcotics and to reduce drug-related harm, *inter alia *"therapy instead of punishment" (1981), substitution-based treatment and distribution of sterile disposable syringes (1992) and medically supervised injection facilities (*drug consumption rooms*) (2000).

Substitution treatment of opiate addicts involves the regular prescription and administration of opiates pursuant to the Narcotics Act. However, the most important mandate is, that in addition to making the required doses of a substitute available, substitution treatment has to consist of a comprehensive and qualified addiction therapy including psychiatric, psychotherapeutic and/or psychosocial measures of treatment and care. Therefore, a close co-operation between physicians and other addiction specialists (e.g. psychiatrists, psychologists, psychosocial counseling services) needs to be realized and individual treatment plans have to be designed for each single patient.

Doctors have to register their patients at the Federal Narcotics Control Board (Bundesopiumstelle) to ensure there is no evidence that a patient receives substitution substances on prescription from another doctor, fails to participate in necessary accompanying treatment and care, uses substances that endanger the purpose of substitution treatment, or uses the substitute in a manner that is prohibited by law (e.g. intravenous use).

Doctors are obliged to document all relevant patient and treatment data. These include case/medical history and results of (physical) examination; indication, diagnosis, treatment goals; formulating of and working towards necessary accompanying support and services (e.g. psychosocial counseling); encoded and anonymous notification of patients to the central substitution register; frequencies and results of drug screenings and supervision of additional use of psychotropic substances; information on the dangers and side effects of collateral substance (mis)use; substitute substance, form, dosage, and dispensing modalities; justification for take-home dosing and current state of treatment; justification for exclusion from treatment; and an individual treatment plan.

Every year, the regional SHIs check a small percentage of documentations by randomly selecting GPs' offices. Substitute substances must not be prescribed for parenteral (intravenous) use. The substitutes prescribed may be dispensed and/or taken under supervision in GPs' offices, hospitals, pharmacies or other facilities approved by the relevant state authorities. Take home medication for up to seven daily doses is possible when the determination of the maintenance dose has been settled and when there is no noxious and/or intravenous concomitant use of other psychotropic substances. Regarding international travels up to 30 take home doses are allowed to be prescribed within a period of 12 months. There are no regulations regarding the minimum age of the patients.

All doctors seeking to provide drug-substitution treatment need special authorization issued by the relevant regional medical boards, and they must provide evidence of having participated in pharmacology and drug addiction training programmes. Training courses are organised by the regional medical boards and span 50 to 60 hours. They cover topics such as opioid dependence and the role of substitute medication, understanding and caring for the substitution patients, assessment and management, and other aspects of clinical practice [12].

### Financing of substitution treatment

Until 2004 SHI funded patients and most patients supported by social welfare had to suffer from illnesses in addition to drug addiction itself to be eligible for substitution treatment. Since then it is sufficient to be diagnosed as being addicted to heroin. In general practice drug users will be accepted for treatment when there is a documented history of compulsive opioid use of two years (according to SHI) and when they are at least 18 years old.

Despite the fact that the SHI guidelines are effective nationwide there are variations among the federal states with respect to the organization and delivery of substitution treatment and accompanying psychosocial care. Depending on the number of substitution treatment providers in a given area doctors can be authorized to treat up to 20 patients or more funded by (SHI). There is no such limitation specified in the Regulations on the Prescription of Narcotics. Thus doctors approved to treat 20 SHI patients may care, for example, for another 20 patients funded by social welfare or an unlimited number of patients who pay for treatment and medication on their own.

### Guidelines of the Federal Medical Board on substitution treatment – improvement of quality of substitution treatment

The guidelines of the German Medical Association on the substitution treatment of opiate addicts, effective since March 2002, specify that treatment is indicated in cases where:

• a manifest opiate dependency is of long standing and attempts at achieving abstinence have not been successful,

• substitution treatment offers the best chance of healing or improvement when compared with other treatment options.

The aim of substitution treatment is to stabilise the drug addicts' health status and gradually move them towards abstinence. It is essential that the accessibility and quality of substitution treatment be further improved. Alongside the implementation of the measures hitherto envisaged for this purpose it is particularly important to:

• improve the psychosocial, psychiatric and psychotherapeutic measures for providing treatment and care and to offer them in sufficient quantities,

• set up quality circles on substitutive therapy at the municipal level.

Substitution treatment is an essential pillar of the treatment of opiate addicts in Germany. To improve quality assurance of MMT the regional medical board of Westfalen-Lippe launched a 'manual on outpatient substitution treatment of opioid addicts'. The manual describes how to define quality and how to ensure that all everyday measures and services are of high quality. The key measures discussed are medical and nursing activities (assessment, diagnosis, documentation, provision of dosage, supervision). This manual is widely used (by 1,000 GPs) as a basis for ensuring good quality in substitution treatment [13].

### Provision of treatment and treatment goals

Various forms of treatment organizations have been developed in Germany. Out-patient counseling services offer contact, motivational and out-patient treatment, whereas detoxification is generally carried out in so-called "regular hospitals" or in a few specialized institutions. In Germany, detoxification is generally carried out in in-patient treatment settings, although there is evidence that out-patient detoxification is also working. There are various kinds of institutions caring for opioid addicts during the phase of rehabilitation, e.g. specialized units at hospitals, specialized clinics or therapeutic communities. In the course of further treatment and after care a wide range of assistance is offered depending on the addicts' needs (concerning, for instance, job finding, housing projects or life in communities). Experts who have generally qualified in specific further education work in these special fields. [14]

The aim of all these offers is to stabilize health and, in the long run, abstinence from drugs. Substitution is the only field which offers non-drug-free treatment. However, substitution is a method which reaches remarkably more drug addicts than any other approach of addiction treatment. So far the linking of the regular system of health provided in Germany and the special addiction treatment system to an efficient unity has not been completely satisfying though co-operation and co-ordination at a regional level are partially well developed.

One of the main standards in drug addiction treatment is the co-operation of different professions including social work/education, psychology and medicine. Operators of centres, the Federal Laender or municipalities, are responsible for quality management and professional supervision of out-patient services.

In contrast to a strong abstinence orientation in the early 1990s the treatment goals are now realistic and pragmatic, such as:

• to assist the patients to stay healthy until, with the appropriate care and support, they can achieve a life free of drugs

• to reduce the use of illicit and non-prescribed drugs by the individual

• to deal with the problems related to drug use

• to reduce the dangers associated with drug use, particularly the risk of death by overdose and HIV and hepatitis infections from injecting and sharing injecting paraphernalia

• to reduce the duration of episodes of drug use

• to reduce the chances of future relapse to drug use

• to reduce the need for criminal activities in order to finance drug use

• to improve overall personal, social and family functioning

Addicts seeking to cope with their addiction with professional support are offered a wide range of assistance approaches to step out of drug use, and there are many therapeutic services available.

Even persons participating in substitution treatment can occasionally be motivated to move on to abstinence therapy. Therefore, a strong co-operation between non-institution doctors and inpatient as well as outpatient addict-support services is necessary to facilitate steps towards abstention from drugs. Furthermore, inpatient drug-therapy facilities must provide slots offering a substitution introductory phase with subsequent abstinence treatment.

Meanwhile, first specialised clinics have been established which also admit clients who are on substitution treatment with the aim of achieving and stabilising abstinence in the course of treatment. Preliminary results show that the success rates of such clinics do not lag behind those achieved by abstinence-oriented therapy. [15]

### Expanding services to improve occupational integration

Regarding employment, the labour market is not easy to access for patients participating in drug-substitution treatment, due to a high general unemployment rate in Germany (10.8% in March 2006 = nearly 4.8 million jobless people) and negative attitudes and beliefs towards the patients on the part of employers. Also, the socio-demographic and biographical characteristics of patients in substitution treatment (e.g. minor school and vocational qualifications, criminal records) reduce the chances of getting employed. Though there are educational and vocational projects in several major cities accompanying support regarding education and employment is still not sufficiently available.

Unemployment is associated with processes of impoverishment, resulting in a large number of psychosocial risks which can have a reinforcing effect on drug use and on the development of substance-related addiction. This is why the German government and treatment services lay great store by the integration into society of persons addicted to psychotropic substances through work and gainful employment. Binding agreements and governing co-operation in the rehabilitation phase are critical between the organisations providing medical and occupational rehabilitation. A great deal of development work awaits the addict support services in this area in the future.

Additional work structures which have to reflect the different capabilities and resources of patients are still missing. Some patients have never functioned in traditional working settings and have to learn to follow certain demands and structures from the very beginning. Others do already have work experiences but are no longer familiar with the demands of the working environment. Work for a certain amount of hours in a charitable institution may constitute one way to solve the problem for a few patients, but many more options are needed to fill the free time and to be effective. [16]

### Promotion and qualification of self-help activities

Self-help groups (including parental self-help groups) should be included to a greater degree in the co-ordinating and planning activities surrounding measures to reduce the problems which arise in dealing with psychoactive substances. They are an indispensable component of the support offered persons who are at risk of addiction or already addicted.

A landmark in the development of self-help activities has been the growing of self-organisation of people who are affected both by drug use and HIV. The opening up of the health sector for self-help and the recognition of the competence of those affected, thanks to the AIDS-Help movement, has led to a new orientation of the somatically focussed medical system in Germany, or at least to first steps in this direction. The self-organisation of people affected in the area of drugs via the development of JES-groups (Junkies, Exusers, Substitute Drug Users) is the most incisive challenge for drug policy and service providers. It requires discussion *with *the people affected and not *about *them. In the meantime, JES groups in nearly 25 cities, with at least some 300 drug users in MMT actively involved, are working as advocates for their own interests. In their founding statement this philosophy is expressed as follows: "JES is a federation based on solidarity among junkies, ex-junkies and substitute drug users who express themselves with the competence of those directly affected, and demands recognition of their existence by state health and drugs policies. Drug users have just as much right to human dignity as everybody else. They do not have to earn this right by abstinence or by conforming. They have a right to humane, healthy and social living conditions." [17]

## Impact of MMT in Germany

### Effectiveness of MMT has been proven

Worldwide, including Germany, methadone maintenance treatment has been evaluated comprehensively [18-21]. On account of different methodological approaches, different evaluation periods and different sample sizes and populations, the German research results are only partially comparable. However, several important common aspects regarding the overall results of German studies and investigations can be presented [4]):

• The average age of methadone patients is above 30 years. The duration of heroin use before starting MMT lies between 10 to 12 years on average.

• More than two thirds of the patients had received treatment in inpatient, drug-free therapeutic communities (TCs) – usually several attempts at treatment – prior to MMT but could seldom complete treatment as expected. One third of the few who left regular therapy immediately relapsed into heroin use.

• MMT shows considerably higher retention rates than TCs (some 65% of clients leave TCs within the first four months of treatment). In North Rhine-Westphalia, for example, the retention rates were 87% after one year, 66% after three years, 53% after five years and 48% after seven years [8]. An evaluation of MMT in Hamburg showed retention rates of 84 % after three years, 77 % after four years, and 71 % after five years [21].

• Even during the initial phase of treatment there is a remarkable improvement in the general health status of methadone patients. The health status of patients infected with HIV or hepatitis also stabilises in the course of treatment. HIV seroconversion rates are well below 1% per year during MMT.

• The risk of mortality is drastically reduced. The survival rate of methadone patients is three to five times higher than of untreated heroin users.

• There is also a reduction in the use of illegal drugs. The decline in illegal use of opioids comes about in a linear way; final cessation is dependent on the duration of participation in treatment. After one year in MMT positive heroin urinalysis ceases among 80 to 90 % of methadone patients. With increasing length of time in treatment there is also a decline in, or termination of, the additional use of other psychotropic substances. (Additional use of other psychoactive substances is not a reason per se to terminate treatment but to change the treatment regime and to determine reasons for this additional use)

• About 10% of treatment participants become totally abstinent (including methadone) [22]. At present, there are no follow-up studies available on the stability of abstinence. However, experiences so far clearly indicate that methadone treatment (detoxification or maintenance-to-abstinence) which is limited in time usually results in a relapse into illegal opioid use and physical as well as psychological instability [23].

MMT is the best investigated and most effective evidence-based treatment of opioid dependents: "Given the chronic, relapsing nature of opioid dependence and the generally disappointing long-term results of detoxification in combination with relapse prevention, agonist maintenance treatment has become the most important treatment modality for opioid dependence" [24].

### Taking account of the results of the pilot programme on heroin-supported treatment in the further development of addict support systems

Although the implementation of MMT has had very positive results in Germany, a certain percentage of participants do not benefit from his type of treatment. This opened the discussion for a diversification of MMT, especially for refractory opioid dependent subjects (either having dropped out of MMT or non-responders in MMT). The positive results of the Swiss heroin trial paved the way for a randomised clinical trial in Germany. [25]

The results of the scientific evaluation of the German pilot project on the heroin-supported treatment of opiate addicts had been recently evaluated [26]. The findings are to be incorporated into the treatment provided to persons suffering from serious heroin addiction who are failing or who have failed to respond well to MMT. Only those opioid addicts were included for whom methadone maintenance had proven ineffective (often during multiple enrolments) or who had not been in treatment for at least 6 months before being included in the heroin trial.

The study was conducted in 7 German cities and 1,032 patients were included at the study centres from 2003 – 2005. One study group was provided with diamorphine (heroin), the other group with methadone. In addition, both groups received special psycho-social support. The retention rate regarding heroin treatment was 67 % after 12 months, slightly lower than the rates reported in studies in Switzerland and the Netherlands. Of the methadone group, only 39 % completed the study treatment. This is mainly a result of the failure of one third of the randomised patients of the control group to show up and start treatment. It must be considered, however, that, at the 12-month examination, 39 % of the dropouts of the heroin group and 44 % of the dropouts of the methadone group were either still in maintenance treatment outside the study conditions or in other addiction treatment settings.

What are the main results of the study? The group of severely ill heroin addicts was successfully recruited. The response-definition was an improvement by 20% in health, a considerable decrease in street heroin consumption and no increase in cocaine use. After 12 months heroin treatment showed significantly better results with respect to improvement in health and the reduction of illicit drug use than methadone treatment. The effects were largely independent of the target group, psychosocial intervention forms and study centre. There was a reduction of cocaine use in both groups. The study demonstrates that heroin treatment can be safely and effectively implemented. No study-related death was reported. The mortality rate was equal in both groups, all death cases were due to previous illnesses. However, higher safety risks in the heroin group (because of injection of the substance) call for treatment in special out-patient clinics and seem to preclude take home medication. Heroin treatment was significantly better than methadone treatment to the group of long term drug users who had previously failed to obtain much benefit from MMT and other forms of treatment with respect to improvement in health and decrease of illicit drug use. As an important additional value, heroin prescription led to a considerable reduction in drug related crimes.

### Psychosocial support – patients' expectations and experiences

The regulations of substitution treatment in Germany demand mandatory participation of patients in psychosocial care, although there is no empirical evidence of a general necessity of psychosocial support for *all *patients [27]. However, these regulations do not provide any instructions on the frequency, mode and scope of psychosocial care provisions and, to date, there are no nationwide standards on how to organize and structure accompanying support. Psychosocial care is a collective name for a number of different services. These may include, for example, legal advice, managing financial problems (e.g. debts, rents), recreational activities, crisis intervention, (psychotherapeutic) group sessions, assistance with finding accommodation and jobs, and qualifying for school and vocational training. Psychosocial care is not funded by the SHI. There are great variations in psychosocial provision between different states and communities, and variations in quality and funding.

Special cognitive-behavioural interventions might help to reduce additional consumption of psychoactive substances [28]. An alternative strategy is contingency management (CM), in the scope of which patients are given positive reinforcement (e.g. vouchers or take-home dose) for each drug-free urine. A multi-centre randomized trial is under way [29].

Worldwide, there is a lack of qualitative research on the subjective views of patients participating in substitution treatment. The attitudes and views of treatment participants deserve to be studied carefully, because one may assume that the more treatment philosophies, policies and settings are oriented towards patients' needs the more favourable outcomes might be expected. [30,4] There is clear evidence of this from reports of patients: *"The doctors, they only know about the effects and side effects from book, but we are the experts. For instance, the doctor says, that everyone who gets methadone feels the same thing but that's not true." *[31].

Psychosocial counseling can support patients with structuring their life again, based on changed values, because the pressure to find drugs is reduced substantially. However, often there are massive problems revealed which might lead to a state of crisis, because the confrontation with injuries, illnesses and other negative experiences of their past can be very painful. The loss of daily structures (and generally all-consuming) activities focused on financing and consuming drugs, the loss of the euphoric effects of substances like heroin and the consequences of massive illness (dual diagnosis, viral infections) and limited future prospects might often lead to depression. Some patients become apathetic and unable to structure their lives. They, for example, hang around all day long watching TV.

Former social networks no longer have the same function they once had. Keeping a distance from the 'drug scene' and establishing a new life is not easy when meeting 'old acquaintances' at the substitution doctor's office every day. The additional use of alcohol and benzodiazepines might function as a kind of self medication to deal with depression but often has the opposite effect. [32]

Improving family life is not easy without professional support, because family integration plays an ambivalent role. Early childhood family experiences are often 'part of the problem'. Family involvement is crucial for the successful treatment while its dynamics might only be understood and confronted with expert psychological support.

### Provision of substitution treatment

It is estimated that about 90% of substitution patients in Germany receive their medication from doctors in independent medical practice (GPs), i.e. not in clinics. However, these practices are mostly run by specialised teams and the patients are nearly exclusively drug addicts. In a survey from spring 1996 in a West-German region 70% of all SHI approved methadone prescribers (598 physicians) in the area were general practitioners, 20% specialists in internal medicine and 6% psychiatrists [33]. About 50 % of GPs in MMT have up to 10 patients, 40 % up to 40 patients and 10 % more than 40 patients. 78 % of the 61,000 patients registered in 2005 got treatment in specialised out-patient services (with their own psycho-social staff), 20 % in practices which were also treating other patient groups, but offering special services for drug users, and (only) 4 % in 'normal' practices of family doctors. [34].

Endorsed by the umbrella organization of the German Association of Pharmacists substitute substances may be legally dispensed via pharmacies since 1998. Table 1 shows the number of registered patients in substitution treatment in Germany.

**Table 1:** Number of registered patients in substitution treatment in Germany

**Date**	**Number**
1992	1,000
1993	4,500
1994	9,700
1995	13,500
1996	19,000
1998	20,000
1999	25,000
2000	33,000
2002	46,000
2003	52,700
2004	57,700
2005	61,000
2006	65,000

From 1992–2000 estimations and data from Health Insurances and Medical Associations; from 2002–2006 data from the federal registry. No data were available for 2001.

### Substances prescribed

When substitution treatment started in Germany only levomethadone was used as a "substitute" (surrogate substance). Now also methadone, buprenorphine and in particular exceptional cases codeine or dihydrocodeine may be prescribed. LAAM or levacetylmethadol (Orlaam^®^) is no longer used in Germany because of dangerous side effects (life-threatening cardiac disorders) [35,36].

Methadone is the substance most frequently prescribed in substitution treatment. In contrast to other countries, there are two forms of methadone available in Germany, the racemic mixture (d, l-methadone) (only available since February 1, 1994) and levomethadone (l-methadone, L-Polamidon^®^). Apart from MMT methadone is also used during detoxification in approved detoxification units where the doses are gradually reduced over a period of one to three weeks.

Regarding codeine/DHC a follow-up study showed that MMT and codeine/DHC treatment are similarly effective in treatment progress and outcome [37,38]. Nevertheless, codeine no longer plays a role in substitution treatment. Due to a change of law – because of severe problems raised by wide medically uncontrolled spread of 'codeine-juice' – the number of codeine/DHC patients decreased from some 25,000 to 30,000 patients in early 1998 to some 5,000 patients in 2001 and to less than 500 in 2005.

By contrast buprenorphine (Subutex^®^) – the substance was approved for substitution treatment by the Federal Institute for Drugs and Medical Devices (Bundesinstitut für Arzneimittel und Medizinprodukte) in early 2000 – has been provided with increasing frequency. It has been suggested that buprenorphine might be especially useful with pregnant women and low-dosed methadone patients [39]. Buprenorphine also appears to be effective when used in detoxification treatment [40]. A report recently published by a Hamburg clinic on the experiences made with the use of buprenorphine (31% of the cases) in comparison with methadone (69%) in withdrawal treatment suggests indications for both substances. The study was carried out on 800 patients between 2000 and 2004. All in all, no significant difference was found for the retention rate (methadone: 52%; buprenorphine: 59%). During methadone withdrawal treatment, 8 out of 10 patients who had undergone long-term methadone substitution displayed significantly less withdrawal symptoms under buprenorphine. The use of buprenorphine in addicted pregnant patients resulted in considerably reduced or absent neonatal withdrawal symptoms. Treated with buprenorphine instead of methadone the patients reported a clearer and more conscious state of mind which was not experienced as positive by all patients, and psychiatric co-morbidity may have been negatively influenced. As a result of careful approach, especially when changing the substances, there were no cases of overdose emergencies during the period under review [41]. Several studies have shown buprenorphine to be effective in maintenance treatment of opioid dependence [23,42]. However, there are no comparative studies on post-detoxification relapse rates. Table 2 shows the substances used for substitution treatment in Germany.

**Table 2:** Substances used for substitution treatment in Germany

**Substances for substitution**	**2002**	**2003**	**2004**	**2005**
Methadone	72,1 %	70,8 %	68,3 %	66,2 %
Levomethadone	16,2 %	14,8 %	15,0 %	15,8 %
Buprenorphine	9,7 %	13,0 %	15,6 %	17,2 %
Dihydrocodeine	1,7 %	1,2 %	0,9 %	0,7 %
Codeine	0,3 %	0,2 %	0,2 %	0,1 %

Die Drogenbeauftragte der Bundesregierung: Drogen- und Suchtbericht 2006 (*Federal Drug Commissioner: Drugs and Addiction Report 2006)*, Berlin, p.69

In a study from Austria [43] patients receiving slow-release morphine in substitution treatment reported lower rates of additional heroin (22.4 % vs. 35.1 %), cocaine (40,9 % vs. 58.3) and benzodiazepine use (74.1 % vs. 88.9 %) compared to those patients who got methadone. The findings confirm other studies indicating that slow-release morphine might offer an alternative in substitution treatment highly appreciated by patients. To date, however, this substance is not available for substitution treatment in Germany.

A 'gold standard' in substitution treatment should not be concentrated on a certain substance, but on the implementation of the individually used substance into a treatment setting which is based on patient's needs, clear regulations, balanced goals and a good patient-doctor relationship [44].

### Substitution treatment in prisons

Under German law the consumption of narcotic drugs as such is not defined as a criminal offence. However, anyone who possesses narcotic drugs for private use and does not have a written permission for their acquisition, is considered to commit an offence pursuant to the Narcotics Act (so-called personal consumption offence), just as anyone who cultivates, produces and trades with narcotics or otherwise brings them into traffic without any official authorization. For this reason a considerable number of opioid addicts in Germany have experienced prison sentences.

In Germany there are approximately 80.000 prisoners, of whom 25 % are considered to be 'problematic drug users'. Up to 50 % of inmates have experienced the use of illicit drugs (mostly cannabis). According to the German Prison Act, each of the 16 federal states is independently responsible for providing adequate medical care to prisoners. Medical care must comply with the medical standards applied outside the prison system. A great number of inmates have a history of injecting drug use and a certain percentage of whom is, although less frequently, still continuing injecting opioids while in prison. Despite rigid controls, it is estimated that 50 % of imprisoned intravenous drug users continue drug taking while in prison [45]. This is associated with high risks of HIV and hepatitis infection transmitted by sharing injecting equipment: sterile syringes and needles are rarely available in prisons. Given these facts there is clearly an opportunity to implement preventive measures within the prison system. Drug treatment can be effective if it is based on sufficient length and quality and continuing aftercare. Meanwhile, in accordance with the WHO "Guidelines of HIV and AIDS in Prisons (WHO, 1993) [46], which recommend that "prisoners on methadone maintenance prior to imprisonment should be able to continue this treatment while in prison", substitution treatment is available in prisons in Germany. However, the implementation is the responsibility of each of the 16 federal states (Laender) and even varies from prison to prison. There are several important distinctions from the services outside the prison system [47,48]. Inmates as patients have no right to choose their doctors; it is not possible to dissociate the patients from the specific intramural inmate 'drug scene', and often there is a lack of positive attitude of the staff towards substitution treatment. Only 6 out of 16 federal states provide substitution treatment in prisons. It is estimated that not more than 700 inmates participate in substitution treatment whereas at least 1/3 of the 10.000 intravenous drug users in prisons on an average day should be eligible for substitution treatment. Admission criteria vary between the states and long-term maintenance treatment is often not an option. Substitution treatment is generally an integral component of a broader drug service concept to reach and stabilise abstinence, to improve access to further treatment after release and to improve relapse prevention. Psycho-social care is provided by social workers from outside the prison, but due to lack of financial resources often falls on prison staff. Sometimes self-help groups (AIDS self-help groups or drug user groups) from outside the prison are allowed to support inmates in treatment. Prison systems are found to be slow in response to epidemics of viral infectious diseases and injecting drug use. However, substitution treatment is known to be an effective response in minimizing the risks and harms of opioid dependent prisoners by reducing heroin use, drug injecting and needle sharing, and prison based drug-trade. The provision should be broadened [49].

### Role of substitution treatment in the treatment of HIV and hepatitis among drug users

Drug users are the second largest risk group of people living with HIV-infections in Germany (the largest group are homosexual men), whereas the group of heterosexual contacts and of people from high-risk countries is quite as large as the drug users' group). According to the Robert-Koch-Institute [50], HIV incidence is at 5.8% (2003:7.0%) among the group of IDU. Until 2000, the figure was 10% and in the mid 1980's there was an incidence rate of 20 %. In some greater cities the percentage of HIV-diagnoses among IDU was about 50 – 60 %. This percentage has dropped significantly due to the implantation of low threshold facilities and MMT. Data from outpatient counseling services show a prevalence of 3.7%. However, it must be noted that recent, large-scale studies allowing for a certain generalization of data are missing.

In summary it may be concluded that intravenous consumption was the probable cause of infection in less than 10% of the new cases and that, in general, less than 5% of the IDU were HIV-positive in the year 2004.

Basic data on viral hepatitis are available for the general population. According to the Federal Health Report [51] 5–8% of the German population in the age of 18–79 years were affected by a hepatitis-B-infection. A total of 0.5–0.7% of the population carries hepatitis-C-antibodies. As for possible ways of transmission, intravenous drug use was mentioned by 7 % among the hepatitis-B cases. In respect to hepatitis-C cases, intravenous drug use at any time in the past was most frequently reported – by 37% of the cases – as the likely route of transmission. In the group of the 20 to 29 year-old male cases, intravenous drug use was reported by 71%. A vaccination study carried out in the open drug scene took a sample of 701 persons finding an antibody prevalence of 38.6% for hepatitis A (anti-HAV), 2.1% for hepatitis B (HBs-Ag) and 34.1% (anti-HBc) as well as 47.5% for hepatitis C (anti-HCV). Only one in five had known they where infected [52]. A survey carried out among 1,512 opiate addicted patients participating in qualified treatment in a Munich clinic, showed the following results (portion of men: 85%, average age 27.7 years, duration of heroin use: 7.8 years, IDU for 6.7 years): hepatitis A was found in 57.7% of the patients, HBV in 33.0% and HCV in 75%. A positive result for HBV and HCV correlated positively with age, duration of intravenous consumption and number of withdrawal treatments respectively [53].

Summarizing, the antibody prevalence (infection rate) of hepatitis B among IDU in Germany can be estimated to range between 40–60% and for hepatitis C between 60–80%. While the data do not permit precise estimates, it is quite obvious that the antibody prevalence in IDU is very high regarding hepatitis B and C.

MMT is the major basis for the treatment of HIV/AIDS-related illnesses among drug users. High retention rates and good compliance with treatment instructions facilitate treatment with antiretroviral medications, either provided in specialised out-patient MMT programmes or in close cooperation with specialised HIV/AIDS hospital units [54]. Long-term substitution programmes allow observation of the course of antiretroviral treatment and a better response to side effects. There are numerous potential drug interactions between antiretroviral medications and methadone and other substitute substances. Adaptations of methadone dosages may be necessary. The analgesic properties of opioids may mask early symptoms of serious side effects of HIV medications. A good relationship between doctor and patient is essential to deal with these problems. In a retrospective study of antiretroviral treatment of drug users (A) and homosexual men (B) in an outpatient specialised centre in Berlin, drug users showed a higher psychiatric co-morbidity, but overall results were similar. After 12 months in treatment the HI-virusload of 44 % of group A was below the limit of detection, compared to 51 % in group B. However, the mortality rate in the drug user group was significantly higher because of deaths due to heart (endocarditis) or liver failure (hepatitis) [55]. Nevertheless, compliance and adherence to ARV (Antiretroviral Therapy) or HAART (Highly Active Antiretroviral Therapy) depend on numerous factors, such as good patient-doctor-relationship, prevalence of psychiatric disorders, level of patients' self-esteem etc.

While in the past IDU had usually been excluded from standard HCV-therapy with Interferon and Ribavirin in Germany, most recent results suggest a different approach. [56,57] Comparisons were made regarding the use of medication in drug users and non-drug users on the basis of the following criteria: response rate, outcome of the HCV-standard therapy as well as severity of neuropsychological side-effects.

• In a controlled prospective study from 2003 [58] no differences were found in persons displaying an addiction related or psychological disorder and a control group without such disorders with regard to psychiatric complications and response rates. However, drug users had a higher drop-out experience.

• In a controlled prospective study from 2004 [59] the treatment outcome of 50 patients in methadone substitution treatment was compared with that of a control group of persons without addiction problems over a period of 5 years. No significant differences were found between the groups, neither for the retention rate nor for the response rate.

• In a group of 40 heroin addicts suffering from severe additional symptoms, [60] response rates were found to be similar to those of the general population.

The results of these studies and other surveys [61,62] suggest that HCV-infected IDU may be successfully treated with a standard therapy. Side effects and response rates correspond with the figures found for the general population. Simultaneous substitution treatment is not an obstacle, but management of both therapies should be closely coordinated [63]. Even in the case of light or moderate additional psychological disorders, HCV-treatment may be carried out successfully, provided it is organized on an interdisciplinary level. [64]. In general, MMT is a prerequisite for a successful additional treatment of HIV or hepatitis in opiate addicted individuals. [65]

## Discussion

After a long and controversial debate methadone maintenance treatment (MMT) was only introduced in Germany in 1987. The number of patients in MMT was low at the beginning because of strict admission criteria, but it has been constantly rising since the 1990s reaching 65,000 at the end of 2006.

One important objective of health policy is to do the utmost to prevent or at least considerably reduce in our society risky and damaging using patterns as well as dependence on addictive substances. Addiction prevention therefore occupies a prominent place in policy efforts. However, it is also an objective to be able to recognize risky consumption patterns at an early stage and reduce them, ensure the survival of those affected and treat cases of dependence with all of the possibilities available according to the current level of scientific knowledge – from abstinence to medically supported therapy. Addiction is a disease that requires treatment.

In Germany, addicts have a legal right to be offered assistance. The bodies responsible for providing social security benefits (the health insurance funds, pension insurance funds, institutions responsible for social assistance, the municipalities) are obliged to finance such assistance. Together with the service-providers and self-help groups, they have succeeded over the past decades in making available a differentiated range of addiction and drug assistance services and facilities which provide addicts in need assistance with a broad spectrum of different support. Over the past 30 years, a high-quality and differentiated treatment system has been developed in Germany in the area of assistance to addicts. This system comprises outreach and low-threshold forms of assistance, outpatient counseling and treatment offers, qualified withdrawal treatment, inpatient detoxification treatment with a subsequent adaptation phase and follow-up, post-inpatient care within the framework of integration (for example: outpatient rehabilitation, special care housing, occupational rehabilitation projects, follow-up care and self-help groups). These offers are supplemented by a medication-assisted outpatient treatment system especially designed for opiate addicts. Co-operation between non-institution doctors and the addict-support system is to be promoted at the interface with acute medicine.

Finally, allocating treatment options (i.e., determining which treatment is best for an individual patient or even for broadly defined subgroups of the addict population) constitutes a key research question [66].

Nevertheless, substitution treatment plays a substantial part in the health care system provided to drug users in Germany.

## Conclusion

In Germany, a history of substitution treatment spanning 20 years has meanwhile accumulated a wealth of experience, e.g. in the development of health care services research; in the development of guidelines and the implementation of quality assurance measures; in the practical implementation of substitution treatment with concomitant effects and treatment elements such as drug-taking history, dosage setting, co-use of other psychoactive substances (alcohol, benzodiazepines, cocaine), management of 'difficult patient populations', integration of the social environment; in the development of programmes designed for psychosocial therapies adjuvant to substitution treatment and, in the framework of the pilot project of 'heroin-based treatment', also with standardised manuals; in allocation research, to find the 'right' therapy form at the 'right' point in time [66]; in the pilot project 'heroin-based treatment' through experience with patients who do not benefit from methadone treatment; through expertise in the treatment of specific co-morbidity such as HIV/AIDS and hepatitis and psychiatric co-morbidity; in the (Europe-wide) use of substitution treatment in prisons; in the promotion and involvement of self-help groups that are highly relevant; in the production, licensing, distribution and control of substitution agents including the setting up of a substitution register; in the framework of the programme *'entwicklungsorientierte Drogenkontrolle' *(development-oriented drug control) of the gtz, the German Agency for Technical Cooperation, in the establishment of alternative development co-operation; in the framework of co-operation with the European Monitoring Centre for Drugs and Drug Addiction (EMCDDA), in the development of Europe-wide standards for substitution treatment.

By combining preventive, therapeutic and repressive measures, drug use should be avoided as far as possible, or, respectively, its consequences should be minimized. To improve the efficiency of public funding for treatment, the co-operation between drug field and standard systems of public help (e.g. youth-oriented help, help for unemployed people) further needs to be developed. Quality assurance of treatment is an important tool of improvement of treatment results. Substitution treatment has become a necessary and the most important part in the treatment process in Germany.

Substitution treatment is widely used, not only in Germany, but in total Europe. After the late 1980s, the rate of MMT accelerated. By 2001, 24 EU countries as well as Bulgaria, Romania and Norway had introduced MMT. Since the mid-1990s also buprenorphine has become a strong part of substitution treatment in Europe [67]. It is estimated that nearly 600.000, more than half of the estimated one million opioid users in Europe, have access to substitution treatment.

But the highest dynamic in the future might be developed in Asia and Eastern Europe. More than half of the world's opiate users are living in Asia and even if the number of drug users per capita is lower than in Europe, the absolute numbers of opioid dependents has been rising dramatically over the past 10 years. It is estimated that currently there are more than 3 m heroin users in China (1.4 m are officially registered), 2 to 4 m opioid users in the Islamic Republic of Iran, several hundred thousand in India and Pakistan, more than 170.000 in Vietnam, several hundred thousand in the Central Asian region, 3 m in the Russian Federation and 380.000 in Ukraine [68]. In nearly all of these countries, apart from Russia which is still rejecting substitution treatment, new substitution treatment programmes mostly providing methadone have been established or are on the way. In the framework of the WHO Collaborative Study on Substitution Therapy of Opioid Dependence and HIV/AIDS in developing countries in Asia and transition countries in Europe [69], convincing results of methadone maintenance treatment had been presented: both the health status and quality of life of the patients participating improved significantly; the severity of dependence clearly decreased by over 50%; also, there was a strong decline in the development of depression; neither HIV nor hepatitis C rates increased. Moreover, high-risk consumption patterns, such as injecting, were reduced significantly. Retention rates were very high after six months, even if the average methadone dose was lower in Asian countries than in Europe. MMT has been shown an effective method for reducing illicit opiate consumption. A rapid expansion of MMT programmes can be expected in the next years.

Future cooperation with European countries, including Germany, will be in demand, not only because of the positive experiences but also to prevent mistakes and failures.

## Competing interests

The author(s) declare that they have no competing interests.
